# Genetic association of lipids and lipid-lowering drug target genes with Endometrial carcinoma: a drug target Mendelian randomization study

**DOI:** 10.3389/fendo.2024.1446457

**Published:** 2024-08-13

**Authors:** Zhehan Yang, Junpan Chen, Minghao Wen, Jiayuan Lei, Ming Zeng, Sichen Li, Yao Long, Zhiyi Zhou, Chunyan Wang

**Affiliations:** ^1^ Department of Obstetrics and Gynecology, Center for Reproductive Medicine, Guangdong Provincial Key Laboratory of Major Obstetric Diseases, Guangdong Provincial Clinical Research Center for Obstetrics and Gynecology, Guangdong-Hong Kong-Macao Greater Bay Area Higher Education Joint Laboratory of Maternal-Fetal Medicine, The Third Affiliated Hospital of Guangzhou Medical University, Guangzhou, China; ^2^ Key Laboratory for Reproductive Medicine of Guangdong Province, The Third Affiliated Hospital of Guangzhou Medical University, Guangzhou, China; ^3^ Department of Clinical Medicine, The Third Clinical School of Guangzhou Medical University, Guangzhou, China; ^4^ The Sixth Clinical Medical School, Guangzhou Medical University, Qingyuan People’s Hospital, Qingyuan, China

**Keywords:** endometrioid carcinoma, mendelian randomization, APOB, CETP, drug target

## Abstract

**Background:**

Aberrant lipid metabolism is intricately linked to the development of endometrial cancer, and statin lipid-lowering medications are regarded as promising adjunctive therapies for future management of this malignancy. This study employed Mendelian randomization (MR) to explore the causal association between lipid traits and endometrial cancer while assessing the potential impact of drug targets on lower lipids on endometrial cancer.

**Method:**

Two-sample Mendelian randomization was employed to probe the causal association between lipid traits and endometrial carcinoma. Drug-target Mendelian randomization was also utilized to identify potential drug-target genes for managing endometrial carcinoma. In instances where lipid-mediated effects through particular drug targets were notable, the impacts of these drug targets on endometrial carcinoma risk factors were investigated to bolster the findings.

**Result:**

No causal association between genetically predicted lipid traits (LDL-C, TG, TC, and HDL-C) and EC was found in two-sample Mendelian randomization. In drug target Mendelian randomization, genetic modeling of apolipoprotein B (APOB) (OR [95%CI]=0.31, [0.16-0.60]; *p*=4.73e-04) and cholesteryl ester transfer protein (CETP) (OR [95%CI]=1.83, [1.38-2.43]; *p*=2.91e-05) genetic mimicry was associated with non-endometrioid carcinoma.

**Conclusion:**

The results of our MR study revealed no causal association between genetically predicted lipid traits (LDL-C, TG, TC, and HDL-C) and EC. Among the six lipid-lowering drug targets, we observed a significant association between lower predicted APOB levels and higher CETP levels with an increased risk of endometrioid carcinoma. These findings provide novel insights into the importance of lipid regulation in individuals with endometrial carcinoma, warranting further clinical validation and mechanistic investigations.

## 1 Introduction

Endometrial carcinoma (EC) is a malignancy originating from the epithelium of the endometrium, which is a prevalent tumor in women and poses a severe threat to their physical well-being (1). In recent years, there has been a global increase in its incidence and disease-associated mortality rates. Historically, EC has been classified into Type I (endometrioid carcinoma) and Type II (non-endometrioid carcinoma, NEC). Type I accounted for about 90% of EC, predominantly comprising low-grade cells that are more prevalent and exhibit a favorable prognosis. Conversely, Type II accounted for about 10% of EC and mainly consisted of high-grade cells that are less common with an unfavorable prognosis (2).

Modern epidemiological research indicates a frequent association between EC and obesity, hypertension, and diabetes, collectively known as the metabolic triad of EC. Studies have demonstrated that individuals with diabetes face a 2.12-fold increased risk of developing EC compared to those without diabetes. Similarly, overweight individuals (BMI ≥ 25 kg/m^2^) have a 2.45-fold higher risk of EC compared to those within normal weight ranges. Moreover, obese hypertensive individuals are at a 3.5 times higher risk of EC compared to their counterparts without these conditions (3). Notably, EC is strongly correlated with metabolic disorders (4).

Dysregulated lipid metabolism, one of the hallmark features of tumors, has garnered increasing attention from researchers in recent years (5, 6). The intricate interplay between lipid traits and EC has garnered significant attention recently, fueled by evidence suggesting a potential causal relationship (7). Diandra et al. reported that dyslipidemia can promote tumorigenesis and immune escape in breast cancer (8), while Carmen et al. found an association between dyslipidemia and ovarian cancer (9). These findings highlight the importance of investigating the role of lipid traits in the development of gynecological malignancies, including EC.

Furthermore, studies have demonstrated the potential benefits of lipid-modifying interventions in reducing cancer risk. An observational study showed a consistent association between statins, a class of lipid-lowering drugs, and a reduced risk of hepatocellular carcinoma (10), suggesting that statins may have chemopreventive effects. Similarly, a randomized controlled trial (RCT) revealed the benefits of statins for uterine leiomyoma (11), further supporting the role of lipid regulation in gynecological health. Additionally, an analysis demonstrated a significant reduction in gastric cancer (GC) risk with Proprotein convertase subtilisin/Kexin type 9 (PCSK9) inhibitors (12), underscoring the potential of lipid-lowering therapies in cancer prevention.

Dyslipidemia has also been suggested to be closely linked to EC (13). Despite this established link, the precise molecular mechanisms underpinning the pathogenesis of EC about lipid traits remain unclear (14, 15). This gap in knowledge highlights the need for further research to elucidate the causal relationship between lipid traits and EC and to identify potential therapeutic targets.

Mendelian randomization (MR) employs genetic variations closely associated with exposure factors as instrumental variables (IVs) to establish causal relationships between exposure and outcome factors (16). In contrast to cross-sectional studies, MR is a novel causal inference method that utilizes instrumental variables (IVs), typically represented by single nucleotide polymorphisms (SNPs), to mitigate confounding and bias in observational studies when exploring the causal effects of treatment methods or drugs. This approach enables the inference of causal links between exposure and outcome by simulating a randomized controlled trial (RCT) environment, and it also compensates for the shortcomings of RCTs (17).

In this study, we employed MR to investigate the association between three lipid profiles, triglycerides (TG), total cholesterol (TC), low-density lipoprotein cholesterol (LDL-C), and high-density lipoprotein cholesterol (HDL-C) with EC. Additionally, the relationship between their respective gene targets and EC was explored to elucidate the underlying mechanism of this process. Furthermore, relevant secondary targets and associated pathways were investigated to substantiate our perspective. Our research results fill the knowledge gap in previous studies and provide a promising treatment approach for clinical treatment. Negative results help reduce unnecessary clinical trials, shorten the time for new drug development, and lower drug development costs.

## 2 Materials and methods

### 2.1 Study design

**Figure 1:**
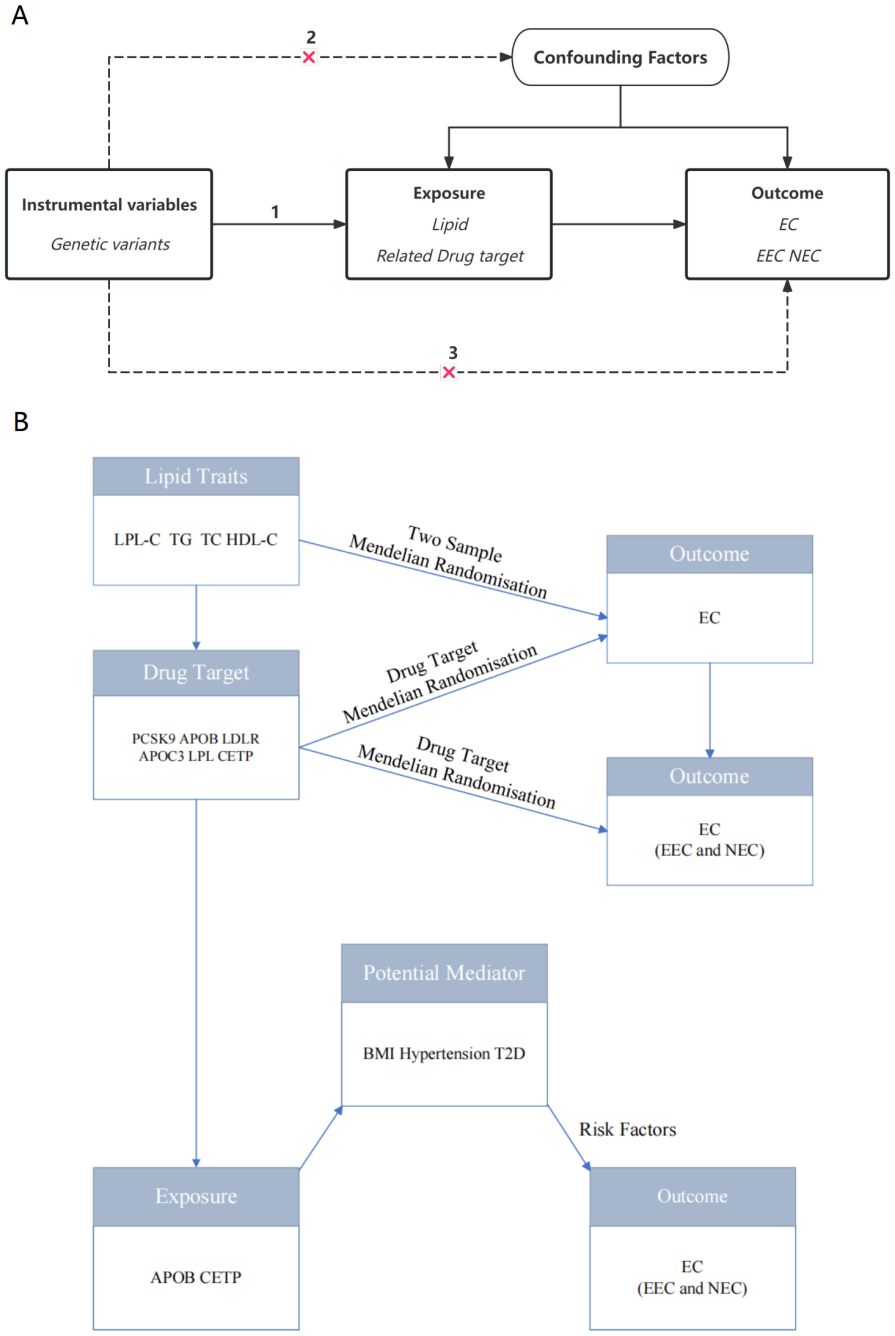
Diagram depicting the study design. **(A)** Flowchart of the research. **(B)** The drug-target MR framework employed in this study relies on three essential assumptions: (1) the selected instrument predicts exposure, (2) The instrumental variables remain independent of confounding factors, and (3) there is no horizontal pleiotropy—the instrument affects the outcome solely through exposure. Abbreviations: LDL-C, low-density lipoprotein cholesterol; TG, triglycerides; TC, total cholesterol; PCSK9, proprotein convertase subtilisin/kexin type 9; APOB, Apolipoprotein B-100; LDLR, LDL Receptor; APOC3, Apolipoprotein C-III; LPL, lipoprotein lipase; CETP, cholesteryl ester transfer protein; EC, endometrial cancer; EEC, endometrioid endometrial cancer; NEC, non-endometrioid endometrial cancer; BMI, body mass index; T2D, type 2 diabetes.

The study was conducted strictly according to the indicators of Reporting Observational Studies in Epidemiology Using Mendelian Randomization (18). The flowchart of this study is presented in **Figure 1A**. The details of the instrument variables are summarized in **Supplementary Table S1**.

### 2.2 The selection of genetic variant

The independent instrumental variables, which were chosen at the genome-wide level (*p*<5×10^−8^), met the criteria of linkage disequilibrium (LD) aggregation threshold (r^2^<0.001) as well as physical distance threshold (10,000 kb). The genetic association estimates were derived for TG, TC, LDL-C, and HDL-C were derived from summary data obtained from GWAS studies, which included a substantial cohort of 115,078 individuals sourced from the website (https://gwas.mrcieu.ac.uk/). The details of the GWAS mentioned above data are presented in **Supplementary Table S2**.

Based on drug target databases and previous studies, six lipid-lowering drug targets have been identified, namely proprotein convertase subtilisin/kexin type 9 (PCSK9), Apolipoprotein B (APOB), LDL Receptor (LDLR) for lowering LDL-C, Lipoprotein Lipase (LPL), Apolipoprotein C-III (APOC3) for lowering TG, and cholesteryl ester transfer protein (CETP) for lowering HDL-C (19, 20) (**Table 1**).

**Table 1:** Gene regions and corresponding positions used for diffierent drug targets.

Primary pharmacological action	Drug-target	Gene	Gene position (GRCh37/hg19)	Number of SNPs
Lowering LDL-C (Primary Pharmacological Action)	Apolipoprotein B-100	APOB	chr2:21,224,301-21,266,945	20
	Proprotein convertase subtilisin/kexin type 9	PCSK9	chr1:55,505,221-55,530,525	27
	LDL Receptor	LDL-R	chr19:11,200,139-11,244,496	40
Lowering TG (Primary Pharmacological Action)	Lipoprotein Lipase	LPL	chr11:116,700,623-116,703,788	11
	Apolipoprotein C-III	APOC3	chr8:19,796,764-19,824,770	15
Lowering HDL-C (Primary Pharmacological Action)	cholesteryl ester transfer protein	CETP	chr16:56,995,862-57,017,757	38

SNPs, single-nucleotide polymorphisms; chr, chromosome; LDL-C, low-density lipoprotein cholesterol; TG, triglyceride.

To estimate drug target exposure, we identified single-nucleotide polymorphisms (SNPs) within a 100 ± kb genomic region of the target gene based on previous literature with a genetically significant threshold (*p*<5×10^-8^) (20). Additionally, to enhance instrumental strength, we employed LD (r^2^ < 0.10 within a 250 kb region) for further selection of the target gene (21). The details of instrumental variables are presented in **Supplementary Tables S3** **S4**.

### 2.3 Outcome of EC

The GWAS data about EC (GWAS ID: ebi-a-GCST006464) were extracted from a dataset encompassing 12,906 cases and 108,979 controls within the European cancer population. Subsequently, these data were further stratified based on histologic type, comprising 8,758 cases of endometrioid carcinoma (EEC) (GWAS ID: ebi-a-GCST006465) and 1,230 cases of non-endometrioid carcinoma (NEC) (GWAS ID: ebi-a-GCST006466) (22). We gathered details of the above EC GWAS data in **Supplementary Table S2**.

### 2.4 Statistical analysis

We employed three analytical methods to investigate the impact of lipid traits and drug targets on diseases: inverse-variance weighted (IVW), weighted median, and MR-Egger. Among these approaches, IVW is widely used and considered the most robust method as it incorporates meta-analysis of multiple-locus effects when analyzing multiple SNPs. All estimates (such as odds ratios [ORs] for EC) are scaled proportionately from the effect of individual SNPs on lipid levels to reflect an increase of 1 mmol/L in lipid levels. The weighted median approach utilizes less than half of the invalid instrumental variables (23). MR-Egger allows for including all instrumental variables under the condition that the basic assumption of MR pleiotropy is not violated (24). The IVW method can provide the most effective causal relationship when all SNPs are valid instruments. However, even if one SNP is invalid, bias in the results may still occur. Therefore, weighted median and MR-Egger are regarded as complementary to IVW in genetic epidemiology studies (25).

Mendelian randomization analysis relies on three critical assumptions: (1) The chosen instrumental variable, typically a genetic variant, demonstrates a strong association with the exposure; (2) The genetic variant shows no association with potential confounders; and (3) The genetic variants affect the outcome exclusively through the exposure and not through alternative pathways (**Figure 1B**).

The Bonferroni correction was employed to account for multiple tests in the analysis. For investigating the effects of lipid traits on EC and six specific lipid targets on EC subtypes, significance levels were adjusted using a Bonferroni-corrected threshold: *P*-values<0.013 (0.05/4), *P*-values<0.008 (0.05/6), and *P*-values<0.004 (0.05/12).

To investigate the potential mediating effects of relevant lipid targets on EC, we evaluated the relationship between lipid-lowering therapies and EC risk factors (BMI, diabetes, and hypertension) using MR analysis. If significant associations are found, it suggests the existence of potential mediation pathways. The BMI dataset was obtained from the Neale Lab Consortium, while genetic estimates related to diabetes were derived from a cohort comprising 468,298 participants (26). The hypertension dataset originated from the FinnGen Consortium (R10). Further details about these datasets can be found in **Supplementary Table S4**.

The F-statistic was employed to evaluate the robustness of each genetic variant (typically, an F-statistic > 10 is considered devoid of instrumental bias). An online tool called mRnd (http://cnsgenomics.com/shiny/mRnd/) was used to calculate statistical power, ensuring it was sufficient. We utilized CHD as a positive control for subsequent validation of significant drug targets. The CHD dataset was acquired from the CARDIoGRAMplusC4D consortium (60,801 cases and 123,504 controls) (19). Cochran’s Q test was applied to detect heterogeneity to ensure further result reliability, while the MR-Egger intercept test and MR-PRESSO were employed to mitigate horizontal pleiotropy. The Steiger test was used to examine the accuracy of causal directionality. Additionally, a leave-one-out approach was implemented to assess the impact of individual SNPs on causality.

The MR results were analyzed using the TwoSampleMR and MRPRESSO packages within R version 4.3.1.

## 3 Results

### 3.1 Lipid traits and EC

**Figure 2:**
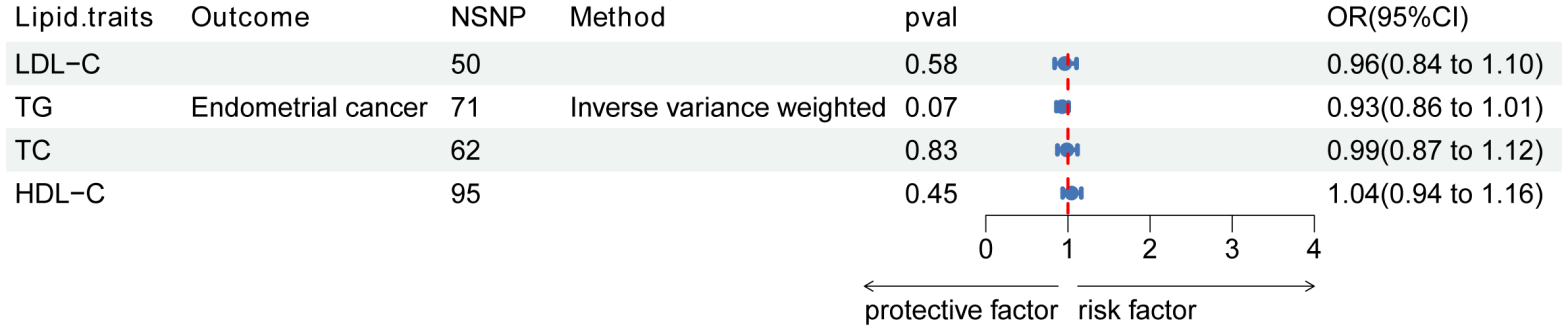
Association of lipid traits with risk of endometrial cancer. Forest plot of the association between LDL-C, TG, and TC with endometrial cancer. Data are presented as ratios (OR) with 95% confidence intervals. Associations are significant after correcting for multiple testing (0.05/4, p<0.013). Abbreviations: LDL-C, low-density lipoprotein cholesterol; TG, triglycerides; TC, total cholesterol; OR, odds ratio; NSNP, number of single-nucleotide polymorphisms.

The instrumental variables for lipid traits comprised fifty independent SNPs associated with LDL-C, 71 SNPs associated with TG, 62 SNPs associated with TC, and 95 independent SNPs associated with HDL-C. However, no significant associations were observed between LDL-C, TG, TC, or HDL-C and endothelial dysfunction (**Figure 2**).

### 3.2 Lipid-lowering drug targets and EC

**Figure 3:**
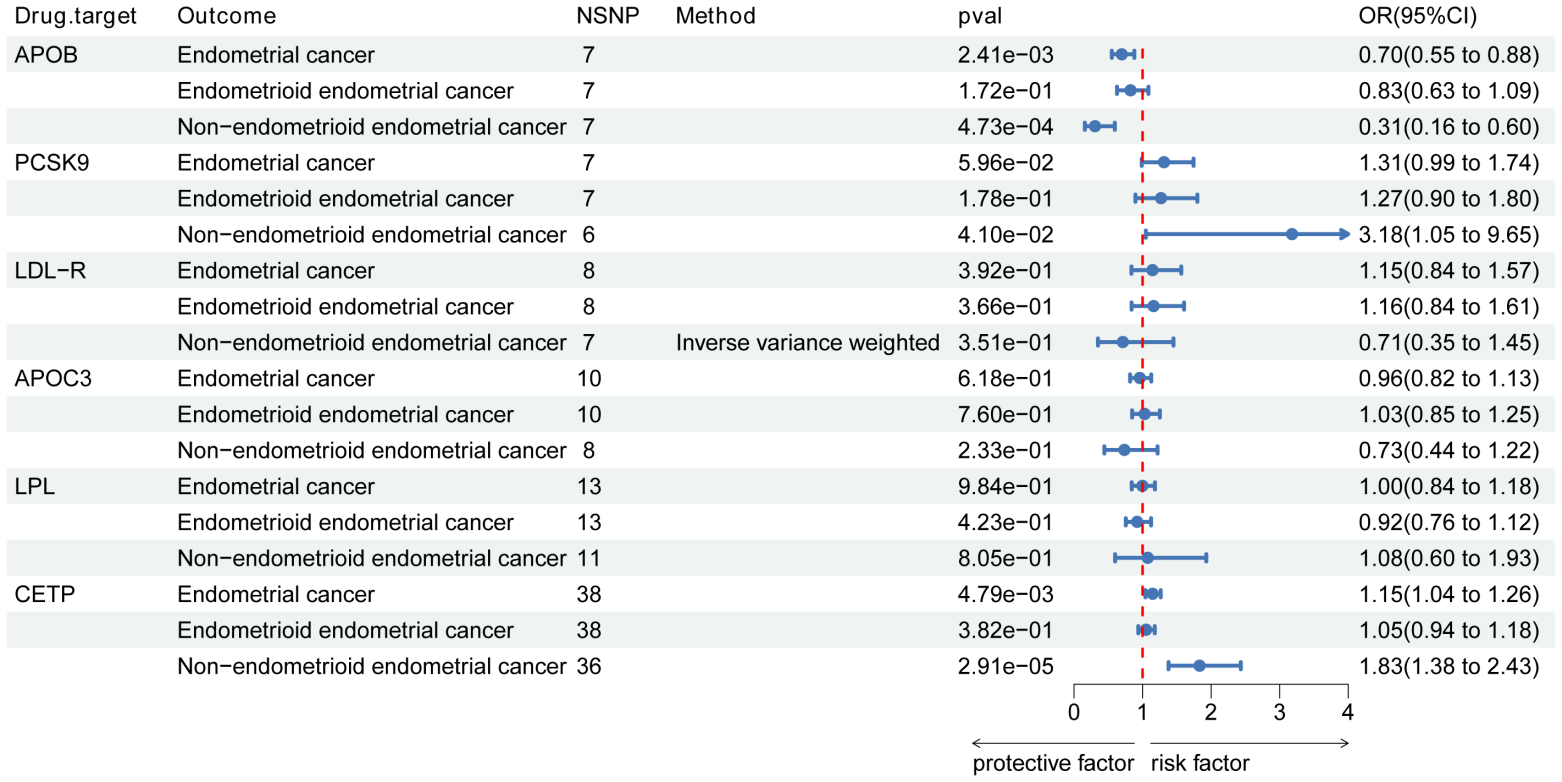
Associations of lipid targets with endometrial cancer and its subtypes. Forest plot of the association between pharmacological targets with endometrial cancer and its subtypes (endometrioid endometrial and non-endometrioid endometrial cancer. Data are presented as ratios (OR) with 95% confidence intervals. Associations of lipid targets with endometrial cancer and its subtypes are significant after correcting for multiple testing (0.05/6, p<0.008 and 0.05/12, p<0.004). Abbreviations: PCSK9, proprotein convertase subtilisin/kexin type 9; APOB, Apolipoprotein B-100; LDLR, LDL Receptor; APOC3, Apolipoprotein C-III; LPL, lipoprotein lipase; CETP, cholesteryl ester transfer protein; OR, odds ratio; NSNP, number of single-nucleotide polymorphisms.

The genetic instruments used in this study included 7 SNPs in APOB, 7 SNPs in PCSK9, 8 SNPs in LDL-R, 13 SNPs in LPL, 10 SNPs in APOC3, and 38 SNPs in CETP (**Figure 3**). With Bonferroni correction, we observed that APOB was associated with a protective effect against EC (OR [95%CI]=0.70 [0.55-0.88]; *p*=2.41e-03), while CETP was identified as a risk factor for EC (OR [95%CI]=1.15 [1.04-1.26]; *p*=4.79e-03).

### 3.3 Lipid-lowering drug targets and EC subtypes

In the analysis of EC subtypes, a significant causal effect was only observed in NEC (**Figure 3**). APOB was identified as a protective factor against NEC (OR [95%CI]=0.31, [0.16-0.60]; *p*=4.73e-04), while no association with EEC was found. CETP, on the other hand, emerged as a risk factor for NEC (OR [95%CI]=1.83, [1.38-2.43]; *p*=2.91e-05) but showed no association with EEC. However, after the Bonferroni correction, we found no specific associations between other lipid targets and EC subtypes. **Figure 3** illustrates the estimated causal effects of exposures on EC and its subtypes.

### 3.4 Significant targets and EC risk factors

In the MR analysis investigating the causal relationship between APOB or CETP and risk factors for EC, our findings suggested that APOB is positively associated with increased BMI, indicating a potential risk factor. At the same time, it exhibits a protective effect by negatively associating with hypertension and T2D (**Table 2**). Moreover, we observed a causal association between CETP and an increase in BMI (**Table 2**). However, no causal relationship was found between CETP and hypertension or T2D.

**Table 2:** Causal relationship between significant targets and risk factors for endometrial cancer.

Outcomes	Exposure	Number of SNPs	Method	OR (95%CI)	P
Body mass index	APOB	6	Inverse variance weighted	1.08(1.04 to 1.13)	<0.001
Hypertension		6	Inverse variance weighted	0.83(0.76 to 0.91)	<0.001
Type 2 diabetes		6	Inverse variance weighted	0.99(0.98 to 0.99)	<0.001
Body mass index	CETP	35	Inverse variance weighted	1.03(1.01 to 1.05)	<0.001
Hypertension		37	Inverse variance weighted	0.97(0.93 to 1.00)	0.073
Type 2 diabetes		36	Inverse variance weighted	1.00(1.00 to 1.00)	0.307

APOB, Apolipoprotein B-100; CETP, cholesteryl ester transfer protein; OR, odds ratio.

## 4 Discussion

Endometrial carcinoma poses a significant threat to women’s health and quality of life. However, the pathogenesis of EC remains elusive. In 1983, Bokhman proposed a strong association between Type I cancers and factors such as prolonged estrogen exposure and increased adiposity. Type II cancers are characterized by higher-grade non-endometrioid tumors, for which the associations above are less significant (27). In this study, we have identified an association between dyslipidemia and Type II EC. Genetically predicted lower APOB levels and increased CETP levels were found to be causally linked to an elevated risk of NEC cases.

Our study did not find evidence regarding the beneficial effects of TG, TC, LDL-C, HDL-C, and other lipid-lowering drug targets on EC. However, previous MR studies have investigated the impact of lipid traits on EC. Pik Fang Kho et al. reported that elevated LDL-C levels were associated with a lower EC risk (OR=0.88 [95%CI, 0.83-0.93]; *p*=7.26×10^-6^), and elevated HDL-C levels were associated with an increased risk of EC (OR=1.07 [95%CI, 1.00-1.14; *p*=0.07), using the LDL-C GWAS dataset from the Global Lipid Genetic Alliance (28). According to Chen et al., LDL-C was negatively correlated with EC risk (OR=0.92, *p*=0.031), and there was no causal link between HDL-C and EC (OR=1.029, *p*=0.474) (29). Both studies identified a correlation between LDL-C and EC risk. However, these studies did not provide strong evidence. At the same time, the discrepancy may be attributed to the confusion caused by linkage disequilibrium(LD) and the inclusion of disparate populations in the study sample (17). In contrast, our study applied stricter LD physical and aggregation thresholds (r^2^<0.001 within a 10,000 kb region) to ensure rigorous screening, which led to the loss of positive results.

More and more studies have shown that dyslipidemia is closely associated with the occurrence and progression of EC, but the underlying etiological link between dyslipidemia and EC remains unclear. Although the exact biological mechanisms and pathophysiological events have not been thoroughly studied, potential mechanisms, including estrogen metabolism, obesity, inflammatory factors, and other interactions, can promote the occurrence and progression of EC. Generalized dyslipidemia encompasses elevated levels of TC and TG, abnormal levels of LDL, ApoA1, ApoB, and other forms of dyslipidemia that are implicated in various processes involving endometrial cancer cells such as growth, proliferation, apoptosis, inflammation, movement, and membrane stability (30). There is still controversy regarding the correlation between lipid profiles and EC. A prospective study including 122 EC patients revealed that low HDL-C levels were associated with an increased risk of EC. However, when obesity was excluded as a factor, the association between HDL-C and EC risk decreased (31). A meta-analysis investigating metabolic syndrome and its components related to EC risk encompassed six studies with 3,132 cases. Subgroup analysis showed a significant correlation between elevated TG levels and EC, while low HDL-C levels did not exhibit a significant association (4).

However, our results failed to establish a causal link between genetically predicted LDL-C, TG, TC, and HDL-C levels and the risk of EC. It is crucial to recognize that the lack of a causal association in our study does not necessarily negate the role of dyslipidemia in EC and underscores the need for more nuanced approaches to understanding the relationship between dyslipidemia and EC. Instead, it may indicate that the relationship is more complex and involves multiple intermediate steps or pathways that were not captured by our analysis. Further research incorporating a broader range of lipid traits, genetic variants, and environmental factors is needed to disentangle the intricate web of interactions contributing to EC risk.

The primary function of ApoB is to participate in the synthesis and secretion of LDL, regulate its clearance rate, and facilitate lipid and cholesterol transportation into tissues (32). Research reports on liver cancer and GC have indicated that pre-treatment levels of ApoB in liver cancer patients are significantly lower compared to those in control groups, suggesting that it may serve as a potential predictor for liver cancer (33). A study investigating lipid metabolism indexes’ relationship with prognosis in GC patients before surgery revealed that the ApoB/ApoA1 ratio level can act as a prognostic factor for GC; higher ratios were associated with poorer overall survival rates among patients. Additionally, elevated levels of ApoB were found to be indicative of a worse postoperative prognosis (34). Furthermore, a study involving 1151 individuals demonstrated that high levels of ApoB may increase the risk for lung cancer and colorectal cancer (35).

However, the unexpected protective effect of a high level of ApoB observed in our study aligns with previous findings in breast cancer research. In a population-based prospective cohort study involving men, we found a positive association between ApoB and cancer risk, while female breast cancer risk showed an inverse relationship with ApoB (35). This discrepancy underscores the importance of considering cancer type, patient demographics, and potential confounding factors when interpreting ApoB’s role in cancer risk and prognosis. The observation that ApoB may have opposing effects on cancer risk depending on the cancer type and patient gender further complicates the picture. The positive association between ApoB and cancer risk in men, coupled with the inverse relationship in female breast cancer, suggests that hormonal and tissue-specific factors may modulate ApoB’s influence. Given the limited research on the correlation between ApoB and EC, as well as the lack of clarity regarding the underlying mechanisms, it is imperative that future studies, particularly multicentre randomized controlled trials, delve deeper into this relationship.

CETP is crucial in maintaining cholesterol balance within cells and the surrounding environment (36). A risky role for CETP was found in other studies of breast cancer, GC, and colorectal cancer. A pilot xenograft mice study corroborated CETP’s function as a cancer survival gene. Suppression of CETP led to a notable inhibition in the growth of triple-negative breast cancer, a breast cancer subtype devoid of hormone receptors, which are typically targeted by endocrine therapies, resulting in an 86% reduction in tumor size (37). In addition, Weimin Wang et al. found that high expression of CETP was worse than low expression in terms of survival of GC patients (38).

Furthermore, a clinical study found increased CETP in colorectal cancer patients (39). Our study found a causal association between increased CETP and EC risk, which is consistent with the effect of CETP on other cancers. Notably, CETP may play an essential role in cancer development and maintenance primarily by affecting the cholesterol balance inside and outside cancer cells. The homeostasis of intra- and extracellular cholesterol not only affects hormone synthesis but is also significant in maintaining cell membranes’ shape and stability (40), implicating CETP as a potential mechanism in EC development. Given the limited research on the relationship between CETP and EC, our speculation that CETP may also function as a survival gene in EC requires further investigation for validation.

Only the predicted APOB and CETP levels were associated with EC in our study. In the EC risk factor analysis, we only found an effect of APOB on the EC triad, while the effect of CETP on BMI may be based on its lipid-lowering effect. The lack of correlation suggests that the APOB gene may have a physiological role in addition to LDL metabolism. Some evidence suggests that hypertension can lead to the development of cancer by inhibiting cell proliferation (41). Furthermore, our results showed a positive correlation between APOB and hypertension, implying a potential mediating role of hypertension between APOB and endometrial cancer. In order to highlight the significant pleiotropic effects of APOB activation, our study links two metabolic co-morbidities of EC (obesity and T2D). APOB-mediated inflammatory effects may play a role in these two combined symptoms (42).

The causal association between APOB CETP and EC in our study bridged a gap in understanding the relationship between lipid targets and EC found in previous research. It offered crucial directional insights for future clinical studies and basic molecular experiments. However, exploring potential mediating effects between CETP and APOB with EC risk factors, while insightful, requires further validation given the potential involvement of additional, unaccounted factors. Additionally, the lack of direct functional validation experiments hinders our understanding of the precise mechanisms by which CETP and APOB may influence EC risk, underscoring the need for in-depth molecular and cellular biology studies.

This research was equipped with some strengths in genetic epidemiology and clinical practice. For genetic epidemiology, it showcases the utility of Mendelian randomization in elucidating causal relationships in complex diseases, overcomes limitations of observational studies, highlights genetic heterogeneity within endometrial cancers, underscores the need for subtype-specific analyses, and identifies potential pathways that can deepen our understanding of NEC’s biological mechanisms. Moreover, Our findings pinpoint APOB and CETP as potential therapeutic targets for NEC, offering prospects for personalized medicine. Future directions include evaluating drug efficacy and safety by targeting these proteins, developing a comprehensive genetic risk score incorporating APOB/CETP variants, conducting prospective cohort studies to assess risk prediction and intervention outcomes, enabling risk stratification and prevention strategies, and ultimately informing public health policies to reduce NEC incidence and mortality through novel preventive and therapeutic approaches.

Several limitations should be taken into account in this study. Firstly, it is essential to acknowledge that gene-environment interactions may influence the phenotype of SNPs as lipid profiles, potentially introducing bias in the experimental results. Moreover, our study employed SNPs as a surrogate for lipid traits rather than directly measuring their values, which could introduce some errors. Genetic heterogeneity cannot be completely ruled out due to inherent population variability. Lastly, given that our study population was limited to European cohorts, caution should be exercised when generalizing these findings to other populations.

## 5 Conclusions

The results of our MR study revealed no causal association between genetically predicted lipid traits (LDL-C, TG, TC, and HDL-C) and EC. However, it is essential to validate these findings using a larger dataset. Additionally, we found that genetically predicted lower APOB levels and increased CETP levels were causally associated with an elevated risk of EC, specifically in NEC cases. This study provides beneficial lipid-regulating agent regimens for EC patients in clinical practice and provides a rationale for the redevelopment of current therapeutic target genes. Nonetheless, further high-quality research is needed to demonstrate the influence of lipid-regulating agents on EC.

## Data Availability

The original contributions presented in the study are included in the article/**Supplementary Material**. Further inquiries can be directed to the corresponding authors.
